# Publisher Correction: Revising the global biogeography of annual and perennial plants

**DOI:** 10.1038/s41586-024-07122-8

**Published:** 2024-01-31

**Authors:** Tyler Poppenwimer, Itay Mayrose, Niv DeMalach

**Affiliations:** 1 School of Plant Sciences and Food Security, Tel Aviv University, Tel Aviv, Israel; 2 Institute of Plant Sciences and Genetics in Agriculture, The Hebrew University of Jerusalem, Rehovot, Israel

**Keywords:** Biogeography, Evolutionary ecology, Plant ecology, Macroecology

Correction to: *Nature* 10.1038/s41586-023-06644-x Published online 8 November 2023

This article was originally published under a standard Springer Nature license (© The Author(s), under exclusive licence to Springer Nature Limited). It is now available as an open-access paper under a Creative Commons Attribution 4.0 International license, © The Author(s). The error has been corrected in the HTML and PDF versions of the article.

