# Supplementary material for: Revising the global biogeography of annual and perennial plants
Source: Nature. 2023 Nov 8;624(7990):109–14. doi: 10.1038/s41586-023-06644-x (PMC10830411; doi:10.1038/s41586-023-06644-x)
Supplement: Supplementary file 1 — Supplementary Notes 1–12, detailing the results of all analyses conducted in the manuscript, information about the databases used and details about the origins of annual and perennial proportion estimates. [file 41586_2023_6644_MOESM1_ESM.docx]

# Supplementary Notes

## Supplement Note 1: Annuals among Herbaceous Species – Results

Here, we present the full results of the analyses presented in the main text for annuals among herbaceous species.

S. Table 1 | Yearly Model Results for Annual Herbs. Results of the multiple linear regression model relating BioClim1, BioClim12, and their interaction to the proportion of annuals among herbaceous species in an ecoregion.

| **Yearly Model**  ***Annuals among Herbs*** | *Estimate* | *Std. Error* | *P-Value* |
| --- | --- | --- | --- |
| (Intercept) | 1.81E-01 | 1.20E-02 | < 1.0^-15^ |
| BioClim1 | 1.12E-02 | 6.41E-04 | < 1.0^-15^ |
| BioClim12 | -7.07E-05 | 1.57E-05 | 8.00E-06 |
| BioClim1  × BioClim12 | -2.13E-06 | 6.80E-07 | 1.81E-03 |
| R-Squared | 0.481 | | |
| DF | 678 | | |
| P-Value | < 1.0^-15^ | | |

S. Table 2 | Quarterly Model Results for Annual Herbs. Results of the multiple linear regression model relating BioClim10, log^­­^_10_ transformed BioClim18, and their interaction to the proportion of annuals among herbaceous species in an ecoregion.

| **Quarterly Model**  ***Annuals among Herbs*** | *Estimate* | *Std. Error* | *P-Value* |
| --- | --- | --- | --- |
| (Intercept) | 0.28 | 0.08 | < 1.0^-15^ |
| BioClim10 | 0.01 | 0.00 | < 1.0^-15^ |
| log10(BioClim18 + 1) | -0.13 | 0.03 | < 1.0^-15^ |
| BioClim10  $\times$ log10(BioClim18 + 1) | 0.00 | 0.00 | 0.49 |
| R-Squared | 0.547 | | |
| DF | 678 | | |
| P-Value | < 1.0^-15^ | | |

S. Table 3 | Temperature Variability Results for Annual Herbs. Results of the linear regression relating temperature variability (T_Var) to the proportion of annuals among herbaceous species in an ecoregion.

| **Temperature Variability**  ***Annuals among Herbs*** | *Estimate* | *Std. Error* | *P-Value* |
| --- | --- | --- | --- |
| (Intercept) | 2.77E-01 | 1.41E-02 | < 1.0^-15^ |
| T_Var | -8.44E-04 | 2.31E-04 | 2.71E-04 |
| R-Squared | 0.019 | | |
| DF | 679 | | |
| P-Value | 2.71E-04 | | |

S. Table 4 | Precipitation Variability Results for Annual Herbs. Results of the linear regression relating precipitation variability (P_Var) to the proportion of annuals among herbaceous species in an ecoregion.

| **Precipitation Variability**  ***Annuals among Herbs*** | *Estimate* | *Std. Error* | *P-Value* |
| --- | --- | --- | --- |
| (Intercept) | 5.84E-02 | 1.25E-02 | 3.80E-06 |
| P_Var | 1.00E-02 | 6.75E-04 | < 1.0^-15^ |
| R-Squared | 0.244 | | |
| DF | 679 | | |
| P-Value | < 1.0^-15^ | | |

S. Table 5 | Human Footprint Results for Annual Herbs. Results of the linear regression relating human footprint (HumanF) to the proportion of annuals among herbaceous species in an ecoregion.

| **Human Footprint**  ***Annuals among Herbs*** | *Estimate* | *Std. Error* | *P-Value* |
| --- | --- | --- | --- |
| (Intercept) | 1.75E-01 | 1.11E-02 | < 1.0^-15^ |
| HumanF | 2.69E-03 | 4.76E-04 | 2.43E-08 |
| R-Squared | 0.045 | | |
| DF | 680 | | |
| P-Value | 2.43E-08 | | |

S. Table 6 | All Major Features Results for Annual Herbs. Results of the multiple linear regression relating the features of the yearly and quarterly model, temperature (T_Var) and precipitation variability (P_Var), human footprint (HumanF), and their interactions to the proportion of annuals among herbaceous species in an ecoregion.

| **All together**  ***Annuals among Herbs*** | *Estimate* | *Std. Error* | *P-Value* |
| --- | --- | --- | --- |
| (Intercept) | 2.97E-01 | 9.41E-01 | 7.53E-01 |
| BioClim10 | 4.75E-02 | 4.47E-02 | 2.88E-01 |
| log10(BioClim18 + 1) | -5.35E-02 | 4.07E-01 | 8.95E-01 |
| T_Var | 4.81E-03 | 1.53E-02 | 7.52E-01 |
| P_Var | -8.03E-03 | 4.91E-02 | 8.70E-01 |
| HumanF | 1.17E-02 | 6.04E-02 | 8.47E-01 |
| BioClim10  × log10(BioClim18 + 1) | -2.17E-02 | 1.92E-02 | 2.58E-01 |
| BioClim10  × T_Var | -8.69E-04 | 7.82E-04 | 2.67E-01 |
| log10(BioClim18 + 1)  × T_Var | -3.19E-03 | 6.83E-03 | 6.41E-01 |
| BioClim10  × P_Var | -7.60E-04 | 2.15E-03 | 7.24E-01 |
| log10(BioClim18 + 1)  × P_Var | 1.90E-03 | 2.26E-02 | 9.33E-01 |
| T_Var  × P_Var | -1.37E-04 | 9.25E-04 | 8.82E-01 |
| BioClim10  × HumanF | -1.34E-03 | 2.53E-03 | 5.97E-01 |
| log10(BioClim18 + 1)  × HumanF | -5.94E-03 | 2.52E-02 | 8.14E-01 |
| T_Var  × HumanF | -4.94E-04 | 1.04E-03 | 6.36E-01 |
| P_Var  × HumanF | 1.54E-03 | 3.08E-03 | 6.17E-01 |
| BioClim10  × log10(BioClim18 + 1)  × T_Var | 4.35E-04 | 3.45E-04 | 2.08E-01 |
| BioClim10  × log10(BioClim18 + 1)  × P_Var | 5.76E-04 | 9.83E-04 | 5.58E-01 |
| BioClim10  × T_Var  × P_Var | 2.54E-05 | 3.97E-05 | 5.22E-01 |
| log10(BioClim18 + 1)  × T_Var  × P_Var | 9.03E-05 | 4.29E-04 | 8.33E-01 |
| BioClim10  × log10(BioClim18 + 1)  × HumanF | 7.51E-04 | 1.06E-03 | 4.78E-01 |
| BioClim10  × T_Var  × HumanF | 3.78E-05 | 4.53E-05 | 4.04E-01 |
| log10(BioClim18 + 1)  × T_Var  × HumanF | 2.80E-04 | 4.44E-04 | 5.29E-01 |
| BioClim10  × P_Var  × HumanF | -4.20E-05 | 1.23E-04 | 7.32E-01 |
| log10(BioClim18 + 1)  × P_Var  × HumanF | -5.81E-04 | 1.37E-03 | 6.72E-01 |
| T_Var  × P_Var  × HumanF | -5.69E-06 | 5.75E-05 | 9.21E-01 |
| BioClim10  × log10(BioClim18 + 1)  × T_Var  × P_Var | -1.40E-05 | 1.82E-05 | 4.44E-01 |
| BioClim10  × log10(BioClim18 + 1)  × T_Var  × HumanF | -2.08E-05 | 1.93E-05 | 2.82E-01 |
| BioClim10  × log10(BioClim18 + 1)  × P_Var  × HumanF | 3.44E-06 | 5.46E-05 | 9.50E-01 |
| BioClim10  × T_Var  × P_Var  × HumanF | -2.20E-07 | 2.28E-06 | 9.23E-01 |
| log10(BioClim18 + 1)  × T_Var  × P_Var  × HumanF | -2.56E-06 | 2.58E-05 | 9.21E-01 |
| BioClim10  × log10(BioClim18 + 1)  × T_Var  × P_Var  × HumanF | 4.90E-07 | 1.02E-06 | 6.32E-01 |
| R-Squared | 0.633 | | |
| DF | 649 | | |
| P-Value | < 1.0^-15^ | | |

## Supplement Note 2: Annuals among All Species – Results

Here, we investigated the proportion of annuals among all species rather than among herbaceous plants (as we did in the main text) (Extended Data Fig 1). Similar to the results presented in the main text, we found that ecoregions with lower precipitation and hotter temperatures (i.e., located in the lower-left coordinate of their biome in Extended Data Fig 1D) possess higher proportions of annuals.

This pattern was corroborated using a linear regression model fitting the annual proportion as a function of *mean yearly temperature* and *total yearly precipitation* (*P* < 1.0^-15^, *D.F.* = 719, *R^2^* = 0.34) (Extended Data Fig 1E). As in the analysis presented in the main text, using bioclimatic features that account for temporal variation in climate throughout the year produced a better-fitting model. The regression model that incorporated the *mean temperature of the warmest quarter* and the log-transformed *precipitation of the warmest quarter* (Extended Data Fig 1F & G) accounted for 47% of the observed variance (*P* < 1.0^-15^, *D.F.* = 719) and outperformed the model based on yearly means in terms of information criteria (ΔAICc = 160).

Qualitatively similar relationships between quarterly climate and annual proportions were found in the four most annual-rich families (Asteraceae, Brassicaceae, Fabaceae, and Poaceae). in all families. The explained variation of annual herb proportions ranged from 49% in Brassicaceae to 18% in Fabaceae (in all models *P* < 10^-15^).

We found that increasing climate unpredictability is associated with a higher proportion of annuals for both precipitation variability (*P* < 1.0^-15^, *D.F.* = 720, *R^2^* = 0.18) and temperature variability (*P* < 1.16^-5^, *D.F.* = 720, *R^2^* = 0.03). Incorporating these features into the quarterly model, which included quarterly temperature and precipitation, further improved the model's fit (*P* < 1.16^-5^, *D.F.* = 706, change in *R^2^* from 0.47 to 0.58, ΔAICc = 143). Finally, the impact of human disturbance was positively correlated, albeit weakly, with a higher proportion of annuals (*P* < 0.0103, *D.F.* = 721, *R^2^* = 0.01). Still, adding this variable to the model with climate unpredictability and quarterly temperature and precipitation further improved the explanatory power of the model (*P* < 1.0^-15^, *D.F.* = 690, change in *R^2^* from 0.58 to 0.62, $\Delta$AICc = 35).

S. Table 7 | Yearly Model Results for Annuals. Results of the multiple linear regression model relating BioClim1, BioClim12, and their interaction to the proportion of annuals among all species in an ecoregion.

| **Yearly Model**  ***Annuals among all Species*** | *Estimate* | *Std. Error* | *P-Value* |
| --- | --- | --- | --- |
| (Intercept) | 1.26E-01 | 9.34E-03 | 3.06E-37 |
| BioClim1 | 5.08E-03 | 4.93E-04 | 2.73E-23 |
| BioClim12 | -2.19E-05 | 1.22E-05 | 7.34E-02 |
| BioClim1  × BioClim12 | -2.58E-06 | 5.22E-07 | 9.61E-07 |
| R-Squared | 0.341 | | |
| DF | 719 | | |
| P-Value | 8.69E-65 | | |

S. Table 8 | Quarterly Model Results for Annuals. Results of the multiple linear regression model relating BioClim10, log^­­^_10_ transformed BioClim18, and their interaction to the proportion of annuals among all species in an ecoregion.

| **Quarterly Model**  ***Annuals among all Species*** | *Estimate* | *Std. Error* | *P-Value* |
| --- | --- | --- | --- |
| (Intercept) | 2.03E-01 | 5.73E-02 | 4.19E-04 |
| BioClim10 | 9.41E-03 | 2.18E-03 | 1.86E-05 |
| log10(BioClim18 + 1) | -6.56E-02 | 2.55E-02 | 1.04E-02 |
| BioClim10  $\times$ log10(BioClim18 + 1) | -2.64E-03 | 9.78E-04 | 7.08E-03 |
| R-Squared | 0.472 | | |
| DF | 719 | | |
| P-Value | 3.2E-99 | | |

S. Table 9 | Temperature Variability Results for Annuals. Results of the linear regression relating temperature variability (T_Var) to the proportion of annuals among all species in an ecoregion.

| **Temperature Variability**  ***Annuals among all Species*** | *Estimate* | *Std. Error* | *P-Value* |
| --- | --- | --- | --- |
| (Intercept) | 8.45E-02 | 9.45E-03 | 3.10E-18 |
| T_Var | 6.90E-04 | 1.56E-04 | 1.16E-05 |
| R-Squared | 0.026 | | |
| DF | 720 | | |
| P-Value | 1.16E-05 | | |

S. Table 10 | Precipitation Variability Results for Annuals. Results of the linear regression relating precipitation variability (P_Var) to the proportion of annuals among all species in an ecoregion.

| **Precipitation Variability**  ***Annuals among all Species*** | *Estimate* | *Std. Error* | *P-Value* |
| --- | --- | --- | --- |
| (Intercept) | 2.51E-02 | 8.61E-03 | 3.64E-03 |
| P_Var | 5.68E-03 | 4.58E-04 | 3.60E-32 |
| R-Squared | 0.176 | | |
| DF | 720 | | |
| P-Value | 3.6E-32 | | |

S. Table 11 | Human Footprint Results for Annuals. Results of the linear regression relating human footprint (HumanF) to the proportion of annuals among all species in an ecoregion.

| **Human Footprint**  ***Annuals among all Species*** | *Estimate* | *Std. Error* | *P-Value* |
| --- | --- | --- | --- |
| (Intercept) | 1.05E-01 | 7.78E-03 | 2.15E-37 |
| HumanF | 8.50E-04 | 3.30E-04 | 1.03E-02 |
| R-Squared | 0.009 | | |
| DF | 721 | | |
| P-Value | 0.010278 | | |

S. Table 12 | All Major Features Results for Annuals. Results of the multiple linear regression relating the features of the yearly and quarterly model, temperature (T_Var) and precipitation variability (P_Var), human footprint (HumanF), and their interactions to the proportion of annuals among all species in an ecoregion.

| **All together**  ***Annuals among all Species*** | *Estimate* | *Std. Error* | *P-Value* |
| --- | --- | --- | --- |
| (Intercept) | 5.02E-01 | 6.58E-01 | 4.45E-01 |
| BioClim10 | 1.57E-02 | 3.06E-02 | 6.07E-01 |
| log10(BioClim18 + 1) | -1.64E-01 | 2.84E-01 | 5.63E-01 |
| T_Var | 2.05E-04 | 1.07E-02 | 9.85E-01 |
| P_Var | -2.07E-02 | 3.43E-02 | 5.47E-01 |
| HumanF | 3.42E-02 | 4.17E-02 | 4.13E-01 |
| BioClim10  × log10(BioClim18 + 1) | -8.94E-03 | 1.30E-02 | 4.93E-01 |
| BioClim10  × T_Var | -3.98E-04 | 5.34E-04 | 4.57E-01 |
| log10(BioClim18 + 1)  × T_Var | -6.76E-04 | 4.78E-03 | 8.88E-01 |
| BioClim10  × P_Var | -5.25E-06 | 1.48E-03 | 9.97E-01 |
| log10(BioClim18 + 1)  × P_Var | 9.45E-03 | 1.58E-02 | 5.50E-01 |
| T_Var  × P_Var | 6.42E-05 | 6.51E-04 | 9.21E-01 |
| BioClim10  × HumanF | -2.30E-03 | 1.73E-03 | 1.82E-01 |
| log10(BioClim18 + 1)  × HumanF | -1.17E-02 | 1.74E-02 | 5.02E-01 |
| T_Var  × HumanF | -8.98E-04 | 7.30E-04 | 2.19E-01 |
| P_Var  × HumanF | 1.81E-04 | 2.14E-03 | 9.32E-01 |
| BioClim10  × log10(BioClim18 + 1)  × T_Var | 2.17E-04 | 2.34E-04 | 3.54E-01 |
| BioClim10  × log10(BioClim18 + 1)  × P_Var | 8.83E-05 | 6.74E-04 | 8.96E-01 |
| BioClim10  × T_Var  × P_Var | 1.28E-05 | 2.75E-05 | 6.42E-01 |
| log10(BioClim18 + 1)  × T_Var  × P_Var | -3.37E-05 | 3.01E-04 | 9.11E-01 |
| BioClim10  × log10(BioClim18 + 1)  × HumanF | 9.19E-04 | 7.21E-04 | 2.03E-01 |
| BioClim10  × T_Var  × HumanF | 5.52E-05 | 3.13E-05 | 7.85E-02 |
| log10(BioClim18 + 1)  × T_Var  × HumanF | 3.62E-04 | 3.12E-04 | 2.46E-01 |
| BioClim10  × P_Var  × HumanF | 2.11E-05 | 8.39E-05 | 8.01E-01 |
| log10(BioClim18 + 1)  × P_Var  × HumanF | -2.00E-04 | 9.42E-04 | 8.32E-01 |
| T_Var  × P_Var  × HumanF | 1.85E-05 | 4.03E-05 | 6.46E-01 |
| BioClim10  × log10(BioClim18 + 1)  × T_Var  × P_Var | -6.16E-06 | 1.26E-05 | 6.25E-01 |
| BioClim10  × log10(BioClim18 + 1)  × T_Var  × HumanF | -2.28E-05 | 1.34E-05 | 8.86E-02 |
| BioClim10  × log10(BioClim18 + 1)  × P_Var  × HumanF | -1.05E-05 | 3.70E-05 | 7.78E-01 |
| BioClim10  × T_Var  × P_Var  × HumanF | -1.33E-06 | 1.58E-06 | 4.03E-01 |
| log10(BioClim18 + 1)  × T_Var  × P_Var  × HumanF | -7.31E-06 | 1.81E-05 | 6.86E-01 |
| BioClim10  × log10(BioClim18 + 1)  × T_Var  × P_Var  × HumanF | 6.32E-07 | 7.09E-07 | 3.73E-01 |
| R-Squared | 0.621 | | |
| DF | 690 | | |
| P-Value | 1E-123 | | |

**Supplement Note 3: Spatial Autocorrelation – Results**

Models accounting for spatial autocorrelation incorporated 39 spatial eigenvectors (80.05% of variance explained). The results below are for the proportion of annuals among herbaceous species.

The Yearly Model refers to the model with BioClim1, *mean yearly temperature* and BioClim12, *total yearly precipitation*. The Quarterly Model refers to the model with BioClim10, *mean temperature of the warmest quarter* and BioClim18, *precipitation of the warmest quarter*. Note that BioClim18 is log_10­_ transformed.

The parameter estimates of the yearly and quarterly models show little difference between those models with and without the set of 39 spatial eigenvectors. Similarly, low p-values are associated with each parameter estimate regardless of including the eigenvectors, suggesting negligible differences. These results demonstrate that the yearly and quarterly models are robust to spatial autocorrelation.

S. Table 13 | Yearly Model Spatial Autocorrelation Comparison. A comparison of the results of the yearly multiple linear regression model with and without 39 spatial eigenvectors (80.05% of variance explained) for annuals among herbaceous species in an ecoregion.

| Yearly Model | **With Eigenvectors** | | | **Without Eigenvectors** | | |
| --- | --- | --- | --- | --- | --- | --- |
|  | *Estimate* | *Std. Error* | *P-Value* | *Estimate* | *Std. Error* | *P-Value* |
| Intercept | 0.122 | 0.015 | 2.77E-15 | 0.181 | 1.20E-02 | < 1.0^-15^ |
| BioClim1 | 0.012 | 8.55E-04 | < 1.0^-15^ | 0.011 | 6.41E-04 | < 1.0^-15^ |
| BioClim12 | -6.44E-05 | 1.53E-05 | 2.74E-05 | -7.07E-05 | 1.57E-05 | 8.00E-06 |
| BioClim1  $\times$  BioClim12 | -6.56E-07 | 6.91E-07 | 0.343 | -2.13E-06 | 6.80E-07 | 1.81E-03 |
| R-Squared | 0.67 | | | 0.48 | | |
| DF | 639 | | | 678 | | |
| P-Value | < 1.0^-15^ | | | < 1.0^-15^ | | |

S. Table 14 | Quarterly Model Spatial Autocorrelation Comparison. A comparison of the results of the quarterly multiple linear regression model with and without 39 spatial eigenvectors (80.05% of variance explained) for annuals among herbaceous species in an ecoregion. BioClim18 is log^­­^_10_ transformed.

| Quarterly Model | **With Eigenvectors** | | | **Without Eigenvectors** | | |
| --- | --- | --- | --- | --- | --- | --- |
|  | *Estimate* | *Std. Error* | *P-Value* | *Estimate* | *Std. Error* | *P-Value* |
| Intercept | 0.119 | 0.077 | 1.20E-01 | 0.276 | 0.077 | 3.60E-04 |
| BioClim1 | 0.015 | 2.94E-03 | 9.10E-07 | 0.014 | 2.94E-03 | 4.79E-06 |
| BioClim18 | -0.065 | 0.033 | 5.12E-02 | -0.128 | 0.034 | 2.04E-04 |
| BioClim1  $\times$  BioClim18 | -1.20E-03 | 1.28E-03 | 0.349 | -9.10E-04 | 1.32E-03 | 0.490 |
| R-Squared | 0.67 | | | 0.55 | | |
| DF | 639 | | | 678 | | |
| P-Value | < 1.0^-15^ | | | < 1.0^-15^ | | |

**Supplement Note 4: Alternative Regression Models – Results**

We applied two alternative regression models to the proportion of annual herbs in ecoregions. First, we applied a logit transformation to the proportion of annual herbs (0.01 was added to all annual herb proportions to avoid 0 values) followed by a linear regression (Extended Data Fig 2A & B).

The Yearly Model refers to the model with BioClim1, *mean yearly temperature* and BioClim12, *total yearly precipitation*. The Quarterly Model refers to the model with BioClim10, *mean temperature of the warmest quarter* and BioClim18, *precipitation of the warmest quarter*. Note that BioClim18 is log_10­_ transformed.

S. Table 15 | Results of the Logit Transformed Yearly Model for Annual Herbs. Results of the multiple linear regression model relating BioClim1, BioClim12, and their interaction to the logit transformed proportion of annuals among herbaceous species in an ecoregion.

| Yearly Model  (*Logit Transformed*) | *Estimate* | *Std. Error* | *P-Value* |
| --- | --- | --- | --- |
| Intercept | -1.817 | 0.085 | < 1.0^-15^ |
| BioClim1 | 0.080 | 4.52E-03 | < 1.0^-15^ |
| BioClim12 | -3.45E-04 | 1.11E-04 | 1.92E-03 |
| BioClim1 $\times$BioClim12 | -1.93E-05 | 4.80E-06 | 6.55E-05 |
| R-Squared | 0.457 | | |
| DF | 678 | | |
| P-Value | < 1.0^-15^ | | |

S. Table 16 | Results of the Logit Transformed Quarterly Model for Annual Herbs. Results of the multiple linear regression model relating BioClim10, log^­­^_10_ transformed BioClim18, and their interaction to the logit transformed proportion of annuals among herbaceous species in an ecoregion.

| Quarterly Model  (*Logit Transformed*) | *Estimate* | *Std. Error* | *P-Value* |
| --- | --- | --- | --- |
| Intercept | -0.814 | 0.591 | 0.169 |
| BioClim10 | 0.065 | 0.023 | 3.95E-03 |
| BioClim18 | -0.989 | 2.64E-01 | 1.89E-04 |
| BioClim10 $\times$BioClim18 | 5.89E-03 | 0.010 | 5.61E-01 |
| R-Squared | 0.440 | | |
| DF | 678 | | |
| P-Value | < 1.0^-15^ | | |

Second, we used a generalized linear model (GLM) with a Poisson distribution and an offset to represent proportion data (Extended Data Fig 2C & D). Note that BioClim18 is log_10­_ transformed.

S. Table 17 | Results of the Yearly GLM for Annual Herbs. Results of the generalized linear model with a Poisson distribution relating BioClim1, BioClim12, and their interaction to the an offset proportion of annuals among herbaceous species in an ecoregion.

| Yearly Model  (*Poisson GLM*) | *Estimate* | *Std. Error* | *P-Value* |
| --- | --- | --- | --- |
| Intercept | -1.839 | 0.015 | < 1.0^-15^ |
| BioClim1 | 0.053 | 8.33E-04 | < 1.0^-15^ |
| BioClim12 | -4.67E-05 | 1.79E-05 | 9.12E-03 |
| BioClim1 $\times$BioClim12 | -2.09E-05 | 8.70E-07 | < 1.0^-15^ |

S. Table 18 | Results of the Quarterly GLM for Annual Herbs. Results of the generalized linear model with a Poisson distribution relating the BioClim10, log^­­^_10_ transformed BioClim18, and their interaction to the an offset proportion of annuals among herbaceous species in an ecoregion.

| Quarterly Model  (*Poisson GLM*) | *Estimate* | *Std. Error* | *P-Value* |
| --- | --- | --- | --- |
| Intercept | -0.235 | 0.081 | 3.79E-03 |
| BioClim10 | 5.12E-03 | 3.19E-03 | 0.109 |
| BioClim18 | -0.996 | 0.036 | < 1.0^-15^ |
| BioClim10 $\times$BioClim18 | 0.019 | 1.44E-03 | < 1.0^-15^ |

**Supplement Note 5: Phylogenetic Relatedness (pGLS) – Results**

The results below used each species' median bioclimatic values as the basis for the continuous response variable. The annual life cycle was incorporated as a dummy variable (0 - perennial, 1 - annual). BioClim10 is the *mean temperature of the warmest quarter* and BioClim18 is the *precipitation of the warmest quarter*. Note that BioClim18 is log_10­_ transformed.

S. Table 19 | Results of pGLS for BioClim10. The results of a phylogenetic relatedness analysis to assess the estimated value of BioClim10 for perennials (score of 0) and annuals (score of 1) among herbaceous species.

| **BioClim10** | **Estimate** | **Std. Error** | **statistic** | **P - value** |
| --- | --- | --- | --- | --- |
| **Intercept** | 23.601618 | 7.021844 | 3.3612 | 0.0007775 |
| **Annual** | 2.872388 | 0.091528 | 31.3826 | < 2.2e-16 |
| **R-Squared** | 0.930 | | | |
| **DF** | 20817 | | | |
| **P-Value** | < 2.2e-16 | | | |

S. Table 20 | Results of pGLS for BioClim18. The results of a phylogenetic relatedness analysis to assess the estimated value of log­_10_ transformed BioClim18 for perennials (score of 0) and annuals (score of 1) among herbaceous species.

| **Log­_10_( BioClim18 +1)** | **Estimate** | **Std. Error** | **statistic** | **P - value** |
| --- | --- | --- | --- | --- |
| **Intercept** | 2.5471175 | 0.8652659 | 2.9437 | 0.003246 |
| **Annual** | -0.1887782 | 0.0091123 | 20.7168 | < 2.2e-16 |
| **R-Squared** | 0.961 | | | |
| **DF** | 20817 | | | |
| **P-Value** | < 2.2e-16 | | | |

**Supplement Note 6: Database Access Dates**

The following table indicates the dates the respective data was downloaded.

S. Table 21 | Database Access Dates. The dates each database was downloaded and their respective citation.

| **Database Name** | **Access** | **Citation** |
| --- | --- | --- |
| BIEN | 01 June 2021 | Maitner, B. S., et al. The bien r package: A tool to access the Botanical Information and Ecology Network (BIEN) database. Methods Ecol. Evol. 9, 373–379 (2018). |
| BROT 2.0 | 23 ‎May ‎2021 | Tavşanoğlu, Ç., Pausas, J. G. A functional trait database for Mediterranean Basin plants. Sci. Data. 5, 180135. figshare https://doi.org/10.6084/m9.figshare.c.3843841.v1 (2018). |
| EOL | 24 ‎May ‎2021 | Parr, C. S., et al. The encyclopedia of life v2: providing global access to knowledge about life on earth. Biodivers. data J. (2014). |
| Engemann *et al*., 2016 | 03 ‎June ‎2021 | Engemann, K., et al. A plant growth form dataset for the New World. Ecology. 97, 3243 (2016). |
| Kew Gardens WCSP | 20 July 2021 | WCSP (2021). ‘World Checklist of Selected Plant Families. Facilitated by the Royal Botanic Gardens, Kew. Published on the Internet; http://apps.kew.org/wcsp/ Retrieved July 20, 2021.’  **direct correspondence* |
| LEDA | 01 ‎June ‎2021 | Kleyer, M., et al. The LEDA Traitbase: a database of life-history traits of the Northwest European flora. J. Ecol. 96, 1266–1274 (2008). |
| Taseski *et al*., 2019 | 01 ‎June ‎2021 | Taseski, G. M., et al. A global growth-form database for 143,616 vascular plant species. Ecology. 53, 2614 (2019). |
| TRY | 09 May 2021 | Kattge, J., et al. TRY plant trait database – enhanced coverage and open access. Glob. Chang. Biol. 26, 119–188 (2020). |
| RAINBIO | 06 June 2021 | Dauby, G., et al. RAINBIO: A mega-database of tropical African vascular plants distributions. PhytoKeys. 74, 1–18 (2016). |
| Rice *et al*., 2019 | 21 June 2021 | Rice, A., et al. The global biogeography of polyploid plants. Nat. Ecol. Evol. 3, 265–273 (2019). |
| USDA | 23 ‎May ‎2021 | USDA, NRCS. 2022. The PLANTS Database (http://plants.usda.gov, 05/23/2021). National Plant Data Team, Greensboro, NC USA. |

**Supplement Note 7: World Flora Online (WFO) – Filtering**

The following steps were used to filter unreliable or poorly matched species. This process was used as part of the name resolution using the R package *WorldFloraOnline* v1.7^53^ as the names database.

1. Species with an *Unchecked* or no taxonomic status were excluded.
2. Species with a Fuzzy Distance (matching distance) greater than two were excluded.
3. Species with a taxonomic rank of *subspecies* or *variety* were united with their respective species, and their taxonomic rank was changed to *species*.
4. Species without a taxonomic rank of *species* were excluded.

**Supplement Note 8: CoordinateCleaner – Filtering**

The following steps were used to discard points with erroneous locations and problematic temporal metadata. This process was applied using *CoordinateCleaner* v2.0-18^55^.

1. Data points flagged by the command *clean_coordinates()* using the following capitals
   1. centroids
   2. equal
   3. gbif
   4. institutions
   5. zeros
   6. countries
2. Data points that were flagged by the command *cf_age()* were excluded.

**Supplement Note 9: Gridded System of Polygons – Methods and Results**

In addition to the ecoregion-based system analyses presented in the main text, we repeated the set of analyses using a grid-based system. The grid system was based on a global tessellation of 100km-by-100km square cells using the R package *sf*^66^. First, using a world map provided by the R package *rnaturalearth*^67^, we determined the percent land coverage of each cell and excluded those cells with less than 50% land coverage, resulting in 14,595 cells. Next, we mapped the cleaned GBIF observation data points (cleaned as deteilaed in the main text for the ecoregion-based system), into each of these cells using the R packages *raster* v3.4-13^57^ and *rgdal* v1.5-27^58^. As with the ecoregion system, we followed the procedures used by ^14^, whereby species were only considered "present" in a cell if there were five or more observations. Similarly, to ensure all cells contained sufficient data for analysis, each cell was only considered if 10 or more species were present. This procedure produced sufficient data for 5,934 cells (~40.7% of cells) when examining annual species among all species and 5,824 cells (~40.0% of cells) for annual species among only herbaceous species.

We find that cells dominated by annuals among herbaceous species (hereafter just called annuals within this supplemental note) are rare, with only approximately 11.7% exhibiting an annual proportion of 50% or more (Extended Data Fig 4A & B).

First, we projected the proportion of annuals in each grid cell into Whittaker’s biomes definitions, as represented using a two-dimensional coordinates system of mean yearly precipitation and temperature. The patterns found using the grid-based system were qualitatively similar but were noisier (probably because ecoregions were designed to minimize variability in environmental conditions within each unit and the smaller number of observations within each grid cell relative to the number of observations within ecoregions). As with ecoregions, cells with lower precipitation and hotter temperatures (i.e., located in the lower-left coordinate of their biome in Extended Data Fig 4C) possess greater percentages of annuals.

This pattern was corroborated using a linear regression model that fitted the proportion of annuals as a function of *mean yearly temperature* and *total yearly precipitation*. Although this gridded system model does not account for as much variation (*P* < 10^-15^, *D.F.* = 5821, *R^2^* = 0.39) as the ecoregion system model (*R^2^* = 0.48), it nevertheless shows the same trend (Extended Data Fig 4D compared to Fig 2C). Again, bioclimatic features that account for within-year variation in climate produced a model with a better fit. The regression model that incorporated the *mean temperature of the warmest quarter* and the log-transformed *precipitation of the warmest quarter* (Extended Data Fig 4E & F) accounted for 42% of the observed variance (*P* < 10^-15^, *D.F.* = 5821) and outperformed the annual climate model also in terms of information criteria (ΔAICc = 227).

We also compared the addition of climate unpredictability and human footprint into the models to assess their impact on annual prevalence. We found that increasing climate unpredictability is associated with a higher proportion of annual species (*P* < 10^-15^, *D.F.* = 5821, *R^2^* = 0.19). Similarly, adding this feature to the model that included quarterly temperature and precipitation further improved the model's fit (*P* < 10^-15^, *D.F.* = 5817, change in *R^2^* from 0.42 to 0.44, ΔAICc = 245). Lastly, the impact of human disturbance was positively correlated with a higher proportion of annuals (*P* < 10^-15^, *D.F.* = 5821, *R^2^* = 0.16). Adding this variable to the model with climate unpredictability and quarterly temperature and precipitation further improved the model's explanatory power (*P* < 10^-15^, *D.F.* = 5809, change in *R^2^* from 0.44 to 0.45, $\Delta$AICc = 100).

66. Edzer, P. Simple features for R: standardized support for spatial vector data. R Package Version 1.0-8. https://cran.r-project.org/web/packages/sf/. (2018).

67. Massicotte, P., South, A. rnaturalearth: world map data from natural Earth. R package version 0.3.0. https://cran.r-project.org/web/packages/rnaturalearth/index.html. (2023).

**Supplement Note 10: GBIF Biases – Results**

We assessed the biases in our dataset with regards to GBIF observational data by first examining the spatial distribution of species missing from our dataset followed by analyses to determine the impact of the number of GBIF species and observations on annual and annual herb proportions.

When examining the proportion of species that were found in GBIF (hereafter “GBIF species”) but missing from our dataset in each mapped ecoregion, we found no identifiable regions with disproportionately high missing species (Extended Data Fig 5A). Furthermore, when projecting ecoregions to their respective Whittaker Biomes, we find that no biome missed substantially more species than others before and after the GBIF filtering procedure (Extended Data Fig 5B). Finally, we found that there are very weak correlations between the proportion of GBIF species missing from our dataset and the proportion of annuals among all species (*P* = 1.317^-7^, *D.F.* = 721, *R^2^* = 0.038) or the proportion of annuals among herbaceous species (*P* = 1.182^-6^, *D.F.* = 680, *R^2^* = 0.034).

Second, our analyses indicated that there is no correlation between the total number of present (5+ observations) GBIF species and the proportion of annuals among all species (*P* = 0.71, *D.F.* = 721, *R^2^* < 0.001) or the proportion of annuals among herbaceous species (*P* = 0.45, *D.F.* = 680, *R^2^* < 0.001) (Extended Data Fig 5D & F).

Additionally, we find very weak correlations between the (log_10_ transformed) total number of observations in an ecoregion and the proportion of annuals among all species (*P* = 2.101^-5^, *D.F.* = 721, *R^2^* = 0.025) or the proportion of annuals among herbaceous species (*P* = 0.65, *D.F.* = 680, *R^2^* < 0.001) (Extended Data Fig 5E & G).

**Supplement Note 11: Biases in Missing Phylogenetic Data – Results**

We explored the biases in our data with regard to the phylogenetic distribution of species missing from our dataset. To this end, we compared the number of species in each family for each non-bryophyte family in the World Flora Online (WFO), which contain 449 families (Extended Data Fig 5H).

- Out of these 449 families:
  - For 288 families, less than 25% of the data is missing.
  - For 393 families, less than 50% of the data is missing.
- The mean percentage of missed species in our dataset is 22.2% per family and the median percentage is 16.2%.

**Supplement Note 12: Human Footprint Correlations**

We found weak correlations between human footprint and the bioclimatic features (individually and in combination) used in the main text (Extended Data Fig 6).

BioClim1is the *mean yearly temperature* and BioClim12 is the *total yearly precipitation* which are the features in the Yearly Model. BioClim10 is the *mean temperature of the warmest quarter* and BioClim18 is the *precipitation of the warmest quarter* which are the features in the Quarterly Model.

- BioClim1: (*P* = 1.0^-15^, *D.F.* = 721, *R^2^* = 0.15)
- BioClim12: (*P* = 8.8^-9^, *D.F.* = 721, *R^2^* = 0.04)
- BioClim1 $\times$ BioClim12: (*P* = 1.0^-15^, *D.F.* = 719, *R^2^* = 0.17)
- BioClim10: (*P* = 1.0^-15^, *D.F.* = 721, *R^2^* = 0.13)
- Log10(BioClim18+1): (*P* = 2.2^-4^, *D.F.* = 721, *R^2^* = 0.02)
- BioClim10 $\times$ log10(BioClim18+1): (*P* = 1.0^-15^, *D.F.* = 719, *R^2^* = 0.15)
